# Candidemia: Changing dynamics from a tertiary care hospital in North India

**DOI:** 10.18502/cmm.8.1.9210

**Published:** 2022-03

**Authors:** Garima Gautam, Deepti Rawat, Ravinder Kaur, Masoom Nathani

**Affiliations:** 1 Department of Microbiology, Lady Hardinge Medical College and Associated Hospitals, New Delhi, India

**Keywords:** Antifungal susceptibility, Blood culture, *Candida*, *Candida tropicalis*, non- *albicans Candida*

## Abstract

**Background and Purpose:**

Routine identification of *Candida* species and knowledge of antibiotic susceptibility patterns can prevent diagnostic delays and help clinicians choose appropriate empirical therapies. This study aimed to identify and speciate *Candida* isolates from bloodstream infections and evaluate their epidemiological profile and antibiotic susceptibility pattern in a tertiary care hospital in North India.

**Materials and Methods:**

Blood samples were cultured in the Department of Microbiology of a tertiary care hospital from January 2019 to May 2021, and the samples which showed growth of *Candida* species (spp.) were included in this study. *Candida* isolates were initially characterized by conventional techniques. Further identification and antifungal susceptibility testing were performed using Vitek 2 compact automated system. Data analysis was performed using the SPSS software (Version 25.0).

**Results:**

*Candida* spp. were isolated from a total of 116 blood samples, 60.92% of which belonged to males. The majority (43.10%) of isolates were obtained from 0-1-month-old neonates, followed by infants (16.38%) and children in the age range of 1-17 years (16.38%). Only 6.89% of isolates were obtained from adults older than 18 years. *Candida tropicalis* (26.72%) was the most common species, followed by *Candida pelliculosa* (19.83%), *Candida albicans* (17.24%), *Candida parapsilosis* (14.66%), *Candida famata* (9.48%), and *Candida krusei* (9.48%). Other isolated species included *Candida lusitaniae*, *Candida sphaerica*, and *Candida inconspicua*. Out of 116 isolates, 101 isolates were subjected to Vitek 2 susceptibility testing. Overall, 21.78% (22/101) of Candida isolates were found to be resistant/intermediate. Among *C. albicans* isolates, resistance was observed only against voriconazole (20%) and fluconazole (5%); however, among non- *albicans Candida* species (NAC), resistance was observed against flucytosine (16.04%), followed by fluconazole (14.81%), voriconazole (3.70%), and caspofungin (3.70%).

**Conclusion:**

Non-*albicans Candida* spp. predominated over *Candida albicans* in causing bloodstream infections and were found to be more resistant to antifungals. Continuous surveillance is necessary to monitor changes in epidemiological and resistance patterns.

## Introduction

Candidemia has remained one of the major causes of morbidity and mortality in health care settings worldwide, especially in the pediatric population [ 1 - 3 ]. Despite an increase in awareness about fungal blood-stream infections (BSIs) in recent years, few studies have been conducted on candidemia in India. According to the data provided by the Centre for Diseases Control and Prevention (CDC) and the National Healthcare Safety Network (NHSN), *Candida* species (spp.) ranked 10th among hospital-acquired pathogens and 5th among nosocomial BSI pathogens after *Staphylococcus aureus*, coagulase negative *Staphylococcus*, *Klebsiella spp.*, and *Enterococcus faecalis* [ 4 ].

During the past decade, the incidence of candidemia has increased substantially which is likely to be due to its association with many risk factors, including long-term hospitalization, antibiotic therapy, use of intravascular catheters or  immunosuppressive agents, and underlying diseases, such as malignancy [ 5 - 7 ]. A high prevalence of candidemia has been observed in India due to several attributable factors, such as favorable climatic conditions and a large population of immuno-compromised patients, including diabetics, people with HIV/AIDS, those using steroids, and subjects with antibiotics misuse [ 8 ]. 

The epidemiology of species causing candidemia is dynamic and evolving. Recent studies have reported an increase in the proportion of BSI cases caused by non-*albicans Candida* species (NAC) [ 3 , 8 - 10 ]. This change in epidemiology has been related to increased use of antifungal agents for prophylactic and empirical treatments with many species found to be less susceptible to azoles and costly alternatives (e.g., echinocandins) being used for NAC [ 8 , 9 , 11 ]. Therefore, early and accurate identification of *Candida* is important for the reduction of morbidity and mortality, especially among infants [ 8 ].

Given the increasing importance of candidemia, this study aimed to identify and speciate Candida isolates from BSIs and evaluate their epidemiological profile and antibiotic susceptibility pattern in a tertiary care hospital in North India.

## Materials and Methods

This retrospective observational study was conducted in the Department of Microbiology, Lady Hardinge Medical College and associated hospitals in New Delhi, India, from January 2019 to May 2021.

 Venous blood culture samples collected were inoculated into blood culture bottles at the site of sample collection. Culture bottles were received from all suspected cases of septicemia and various locations, such as intensive care units (ICU), outpatient departments (OPDs), and wards. A single sample was taken from each patient. Samples with the growth of single Candida spp. were included in the study; however, sterile samples and samples with mixed growth or bacterial growth were excluded. Informed written consent was obtained from the patients providing blood samples, and the study protocol was approved by the Ethical Committee for Human Research of Lady Hardinge Medical college and associated hospitals, New Delhi, India (Registration no: ECR/435/Inst/DL/2013; Ethics code:LHMC/ ECHR/2018/43T).

Clinical isolates of *Candida* spp. were obtained from blood culture using the automated culture system BacT/Alert3D (BioMérieux, India) as well as the manual blood culture method. Isolates were subcultured on Sabouraud Dextrose Agar for further work-up. Subcultures were incubated at 37°C for 24 h and another 24-48 h, as required. Initially, the growth obtained was identified by conventional methods and then verified using an automated system. The employed conventional tests included Gram-stain, germ tube test, growth on CHROMagar, and Corn Meal Agar. The automated system used was Vitek 2 (Biomerieux, France). The antifungal susceptibility testing (AFST) was performed using AST-YS08 cards and included minimum inhibitory concentration to fluconazole, voriconazole, micafungin, caspofungin, amphotericin-B, and flucytosine. *Candida krusei* ATCC 6258 was used as a control. 

Data obtained from this study were analyzed using descriptive statistics, such as percentage and proportion. The Chi-square test was used to determine the association of various parameters with *C. albicans* and NAC. All the data analysis was performed using the SPSS software (version 25.0).

## Results

A total of 116 (0.34%) *Candida* spp. isolates were obtained out of 33,445 clinically suspected cases of septicemia, from January 2019 to May 2021. In total, 60.92% and 39.08% of isolates (n=116) belonged to males and females, respectively. The majority of isolates were obtained from 0-1-month-old neonates (43.10%) followed by infants (16.38%) and children in the age range of 1-17 years (16.38%). Only 6.89% of isolates belonged to adults older than 18 years old, and 75% of isolates were taken from patients admitted to wards, followed by those admitted in various ICUs (19.83%) and nurseries (4.31%).

Out of 116 isolates, 20 (17.24%) and 96 (82.76%) isolates were *C. albicans* and NAC species, respectively. Among NAC species, *Candida tropicalis* (n=31, 26.72%) was the most common species, followed by *Candida pelliculosa* (n=23, 19.83%), *Candida parapsilosis* (n=17, 14.66%), *Candida famata* (n=11, 9.48%), and *Candida krusei* (n=11, 9.48%). Other isolated species included *Candida lusitaniae*, *Candida sphaerica*, and *Candida inconspicua*.

Out of 116 isolates, Vitek 2 reported a susceptibility profile of 101 Candida species. Susceptibility patterns of *C. famata* and *C. sphaerica* were not reported by Vitek 2 system. Overall, 21.78% (22/101) of *Candida* isolates were resistant or intermediate (R/I). Out of the 20 *C. albicans* isolates, four (20%) isolates were found to be resistant, whereas 18 (22.22%) isolates out of a total of 81 NAC isolates reported by Vitek 2, were found to be R/I. 

Among *C. albicans* isolates, three (15%) isolates were resistant to voriconazole, and one (5%) isolate was resistant to both voriconazole and fluconazole, whereas, among NAC isolates, the highest resistance was observed against flucytosine (16.04%), followed by fluconazole (14.81%), as well as caspofungin and voriconazole (3.70%). The lowest resistance was observed against amphotericin B, and all the isolates were uniformly sensitive to micafungin. Highly resistant isolates included *C. krusei* and *C. pelliculosa*. Out of all resistant *C. albicans* isolates, 75% of isolates were resistant to a single drug, whereas the majority (96.8%) of NAC isolates were multidrug-resistant. The resistance profile of Candida, based on species, is presented in Table 1. Voriconazole resistance was higher in *C. albicans* (20%) than NAC isolates (3.7%).

**Table 1:** Resistance profile of Candida species to antifungal drugs

Organism	Antifungal Drug											
	Fluconazole		Voriconazole		Caspofungin		Micafungin		Amphotericin B		Flucytosine	
	R	I	R	I	R	I	R	I	R	I	R	I
*C. albicans* n=20, (%)	1 (5)	0	4 (20)	0	0	0	0	0	0	0	0	0
NAC n=81, (%)	12 (14.81)	0	3 (3.70)	0	3 (3.70)	0	0	0	2 (2.46)	4 (4.93)	13 (16.04)	1 (1.23)
*C. tropicalis* n=29, (%)	2 (6.89)	0	3 (10.34)	0	1 (3.44)	0	0	0	0	1 (3.44)	0	0
*C. pelliculosa* n=23, (%)	1 (4.34)	0	0	0	1 (4.34)	0	0	0	1 (4.34)	0	3 (13.04)	0
*C. parapsilosis* n=17, (%)	0	0	0	0	1 (5.88)	0	0	0	1 (5.88)	0	0	0
*C. krusei* n=10, (%)	9 (90)	0	0	0	0	5 (50)	0	0	0	3 (30)	10 (100)	0
*C. inconspicua* n=1, (%)	0	0	0	0	0	0	0	0	0	0	0	1 (100)
*C. lusitaniae* n=1, (%)	0	0	0	0	0	0	0	0	0	0	0	0
Total n=101, (%)	13 (12.87)	0	0 (6.93)	0	3 (2.97)	5 (4.95)	0	0	2 (1.98)	4 (3.96)	13 (12.87)	1 (0.99)

R- resistant, I- Intermediate

In total, 50% and 9.09% of the R/IS isolates were taken from neonates and infants aged 2-11 months, respectively. Moreover, 18.18% of isolates were taken from children aged 1-17 years, whereas only 4.45% of these isolates were from adults older than 18 years. All

the *C. albicans*-resistant isolates were obtained from males, whereas NAC-resistant isolates were higher in females (61.11%), and the difference was found to be statistically significant (*P*<0.05). In total, the distribution of R/I isolates was equal among males (50%) and females (50%). The highest number of R/IS isolates (63.63%) were taken from patients admitted to wards and 27.27% of isolates were obtained from patients in ICUs. It should be noted that all of the isolates obtained from OPDs were sensitive (Table 2).

**Table 2:** Comparison of the epidemiological profile of resistant and intermediate Candida isolates

		*C. albicans* (n=4)	NAC (n=18)	P-value	Total (n=22), n (%)
**Age**	0-1 month	2	9	0.55	11 (50)
	2-11 months	0	2		2 (9.09)
	1-5 years	0	1		1 (4.45)
	6-11 years	1	0		1 (4.45)
	12-17 years	1	1		2 (9.09)
	18-45 years	0	1		1 (4.45)
	> 45 years	0	0		0
	Not available	0	4		4 (18.18)
**Gender**	Male	4	7	0.04	11 (50)
	Female	0	11		11 (50)
**Location**	Ward	1	13	0.12	14 (63.63)
	ICU	2	4		6 (27.27)
	Nursery	1	1		2 (9.09)
	OPD	0	0		0

## Discussion

The genus *Candida* is commensals that normally colonize mucosal and skin surfaces. Mucocutaneous infections (i.e., oral thrush, vaginitis, or skin infections in moist folds) are more common and are responsible for invasive conditions, such as candidiasis and candidemia in hospital-based settings [ 12 ]. Candidemia is one of the most common BSIs and its incidence is on the rise in many countries [ 11 ]. 

In this study, a total of 116 *Candida* spp. isolates were obtained from January 2019 to May 2021. The majority of isolates belonged to infants and children. Similar results were observed in the study conducted by Ahmed et al. (2019), in which the majority of isolates belonged to neonates (47.89%) and infants (25.35%). Increased risk of candidemia in neonates was attributed to an immature immune system, longer ICU stay with exposure to nosocomial pathogens, and the requirement of invasive procedures [ 3 ].

National or multicentric data are lacking from India; however, in individual studies, 6%-8% of candidemia cases have been reported with increasing isolation of NAC species. Among them, *C. tropicalis* is found to be the most common species [ 7 , 11 ] In this study, the majority of isolates were NAC (82.76%) with *C. tropicalis* (26.72%) as the most common species. In a study conducted by Ahmed et al. in 2019, NAC species (n=61, 85.91%) predominated over *C. albicans* (n=10,14.08%)[ 11 ]. Consistently, in a study conducted by Mishra R. (2020), the majority of the Candida isolates obtained from the PICU of an Indian hospital included NAC (86.1%) and C. tropicalis (44.4%) identified as the most prevalent *Candida* species followed by *C. parapsilosis* (16.7%) and *C. glabrata* (16.7%) [ 13 ]. Other studies have also reported *C. tropicalis* and *C. parapsilosis* as the most prevalent Candida species [ 6 , 12 , 14 ]. *C. pelliculosa* was the second most common NAC isolates in our study. It has been reported to be a rare pathogen of fungemia. There have been a few nosocomial outbreaks of *C. pelliculosa* fungemia in nurseries and pediatric ICU and hematologic units [ 15 , 16 ]. *C. glabrata* was not isolated in the present study, though it has been reported as one of the common invasive *Candida* species [ 9 , 13 , 14 ]. The distribution of *Candida* spp. reported in various studies is presented in Table 3. 

**Table 3:** Distribution of Candida species reported in various studies

Study	Year	*C. albicans*, %	NAC, %	Most common NAC (%)
Deorukhkar [ 21 ]	2012	40.2%	59.8%	*C. tropicalis* (26.8%)
Thomas et al. [ 22 ]	2016	14.3%	85.7%	*C. tropicalis* (50.5%)
Bhattacharjee P. [ 14 ]	2016	48.57%	51.42%	*C. tropicalis* (24.28%)
Gandham et al. [ 12 ]	2016	10.34%	89.66%	*C. tropicalis* (34.4%)
Tejan et al. [ 8 ]	2017	20.8%	79.2%	*C. tropicalis* (42.1%)
Divakar et al. [ 6 ]	2017	22.5%	77.5%	*C. tropicalis* (30.8%)
Kumar et al. [ 3 ]	2019	21.3%	78.7%	*C. parapsilosis* (29.8%)
Mishra et al. [ 13 ]	2020	13.88%	86.11%	*C. tropicalis* (44.4%)
Ahmed et al. [ 11 ]	2020	14.08%	85.91%	*C. tropicalis* (35.2%)
Sharma et al. [ 9 ]	2020	10.8%	89.2%	*C. auris* (32.4%)
Ahangarkani et al. [ 19 ]	2020	49.0%	51.0%	*C. guilliermondii* (10.9%)
This study	2021	17.24%	82.76%	*C. tropicalis* (26.7%)

NAC- Non – albicans Candida species

In the evaluation of the global scenario, candidemia is one of the most common bloodstream infections in the United States. Between 2013 and 2017, the average incidence was approximately nine per 100,000 people, with NAC species accounting for approximately two-thirds of candidemia cases [ 17 ]. The frequency of *C. albicans* is decreasing, and studies from Northern Europe and the USA reported a high number of cases caused by *C. glabrata*, whereas studies from Spain and Brazil demonstrated a lower number of cases caused by *C. glabrata* and a higher number of cases attributed to *C. parapsilosis* [ 18 ]. Another study from Iran conducted by Ahangarkani et al. in 2020 showed that *Candida albicans* was the most frequent (49%) causative agent, and all fluconazole-resistant species were NAS species [ 19 ]. These species are now becoming major agents of nosocomial candidemia in high-risk pediatric patients [ 19 ].

The resistance pattern of *Candida* spp. is ever-changing and varies in different geographical areas. In our study, C. albicans was found to be resistant to voriconazole (20%) and fluconazole (5%), and uniformly susceptible to caspofungin, micafungin, flucytosine, and amphotericin B. Resistance to fluconazole and voriconazole has been reported to be as high as 80% and 60%, respectively [ 13 ]. In contrast, one study by Bhattacharjee showed that *C. albicans* was 53.6% resistant to amphotericin B, 64.3% resistant to flucytosine, and only 10.7% resistant to voriconazole, whereas all isolates were susceptible to fluconazole [ 14 ]. *C. albicans* has also been found to be susceptible to all the antifungal agents [ 8 , 9 , 11 ]. 

In this study, NAC was highly resistant to flucytosine (16.04%) followed by fluconazole (14.81%), caspofungin, and voriconazole (3.70%). Similarly, a study conducted by Bhattacharjee in 2019, reported 52.94% resistance to both fluconazole and flucytosine in *C. tropicalis* [ 14 ]. Another study reported 75% fluconazole resistance in *C. tropicalis* [ 13 ]. Another study reported *C. tropicalis* to be only 12.5% resistant to fluconazole, and there was a uniform susceptibility to all other antifungals, including caspofungin and micafungin [ 11 ]. Table 4 presents different resistance patterns of *Candida* spp. all over India. 

**Table 4:** Resistance pattern of Candida spp. reported in various studies in India

Study	*C. albicans*, %				*C. tropicalis*, %				*C. pelliculosa*, %				*C. krusei*, %			
	F	V	A	FL	F	V	A	FL	F	V	A	FL	F	V	A	FL
Deorukhkar, 2012	8.9	-	2.5	-	36.5	-	3.8	-	-	-	-	-	10	-	0	-
Bhattacharjee P., 2016	0	10.7	53.6	0	52.9	29.4	29.4	52.9	0	0	0	0	-	-	-	-
Thomas et al., 2016	47	-	0	-	49.0	-	0	-	-	-	-	-	100	-	0	-
Nazir and masoodi, 2018	42	0	14	28	49.0	0	10	42	-	-	-	-	-	-	-	-
Kumar et al., 2019	30	0	0	-	45.4	9.1	9.1	-	-	-	-	-	100	0	0	-
Mishra et al., 2020	80	60	40	-	75.0	6.2	0	-	100	0	0	0	100	0	0	-
Ahmed et al., 2020	0	-	0	-	12.5	0	0	0	0	0	14.2	0	100	-	0	-
This study, 2021	05	20	0	0	6.8	10.3	0	0	4.3	0	4.3	13	90	0	0	100

F- Fluconazole, V- Voriconazole, A- Amphotericin B, FL- Flucytosine

Fluconazole resistance is on the rise as it is the most widely used antifungal drug empirically given to all high-risk patients, which has ultimately resulted in the development of resistant strains, such as *C. glabrata* and intrinsically fluconazole-resistant strains of *C. krusei* [ 11 , 13 ]. Studies show a marked difference in the behavior of various NAC strains

such as factors that affect the ability to cause disease and develop drug resistance mechanisms. Therefore, the findings in *C. albicans* cannot be applied to the NAC species [ 20 ]. In the present study, voriconazole resistance was higher in *C. albicans* (20%) than in NAC species (3.7%). According to CDC, 7% of all *Candida* bloodstream isolates in the USA have been resistant to fluconazole. More than 70% of these resistant isolates are *C. glabrata* or *C. krusei* species. The fluconazole-resistance pattern has remained fairly constant over the past 20 years, whereas echinocandin resistance appears to be on the rise, especially among *C. glabrata* isolates [ 17 ].

Risk factor analysis is an important aspect of studying candidemia since it helps to decide the application of prophylactic therapy and preventive measures among patients at risk [ 21 ]. Among variables evaluated as risk factors, all the *C. albicans* resistant isolates were obtained from males, whereas resistant isolates of NAC were higher in females (61.11%), and the difference was found to be statistically significant (*P*<0.05). Studies have shown that candidiasis is more common in females than males [ 14 ]. A study performed by Tejan N. in 2017, reported that 90% of subjects in the *C. albicans* group were male, while in the NAC group 57.9% of subjects were female [ 8 ]. This can be explained by the colonization of the female genital tract with NAC which might invade the bloodstream during hospitalization. In our study, the rest of the variables in the resistant group were found to be non-significant.

Regarding the limitation of this study, one can refer to the lack a correlation between resistance patterns and other risk factors, such as antifungal use, immunocompromised status, use of corticosteroids, and organ transplantation, or malignancies. It should be noted that this single-centre study included routine samples received in the microbiology department and might not reflect the epidemiology of species causing candidemia in the whole country.

## Conclusion

The increasing prevalence of candidemia in the Indian setting highlights the need for prior knowledge of local and national species distribution and antifungal susceptibility patterns to tailor the antifungal stewardship program. The changing dynamics of resistance pattern among various *Candida* species requires continuous monitoring of susceptibility pattern. This would facilitate early and accurate diagnosis of the condition and timely initiation of appropriate antifungal therapy. However, further studies with a larger sample size are required to formulate antifungal policies and guidelines for the treatment of candidemia that would ultimately decrease the prevalence of resistant strains.

## Acknowledgments

We thank all our colleagues at the Department of Microbiology, Lady Hardinge Medical College and associated hospitals in India who supported us with their insight and expertise. We thank all the faculty members for assisting in data acquisition and providing comments that greatly improved the manuscript.

## Authors’ contribution

G.G. contributed in the study design, literature search, data analysis, statistical analysis, and manuscript preparation. D.R. and R.K. contributed in concept development, study design, definition of intellectual content, data acquisition, manuscript editing, and review. M.N. contributed in data acquisition and analysis. 

## Conflicts of interest

None.

## Financial disclosure

None.
